# Thrombopoietin-independent generation of platelet-like particles from megakaryoblastic cells

**DOI:** 10.1038/s41598-023-50111-6

**Published:** 2023-12-18

**Authors:** Nuntiporn Nunthanasup, Nutpakal Ketprasit, Egarit Noulsri, Attakorn Palasuwan, Valery Combes, Kasem Kulkeaw, Duangdao Palasuwan

**Affiliations:** 1 Program in Clinical Hematology Sciences, Department of Clinical Microscopy, Faculty of Allied Health Sciences, Chulalongkorn University, Bangkok, 10330 Thailand; 2 Oxidation in Red Cell Disorders Research Unit, Department of Clinical Microscopy, Faculty of Allied Health Sciences, Chulalongkorn University, Bangkok, 10330 Thailand; 3 Research Division, Faculty of Medicine, Siriraj Hospital, Mahidol University, Bangkok, Thailand; 4 Malaria and Microvesicles Research Group, School of Life Science, Faculty of Science, University of Technology Sydney, Ultimo, Sydney, NSW 2007 Australia; 5 Siriraj Integrative Center for Neglected Parasitic Diseases, Department of Parasitology, Faculty of Medicine Siriraj Hospital, Mahidol University, Bangkok, 10700 Thailand; 6 Present Address: Department of Biochemistry and Pharmacology, Bio21 Molecular Science and Biotechnology Institute, The University of Melbourne, Melbourne, VIC 3052 Australia

**Keywords:** Biological techniques, Cell biology

## Abstract

The use of megakaryoblastic leukemia MEG-01 cells can help reveal the mechanisms of thrombopoiesis. However, conventional in vitro activation of platelet release from MEG-01 cells requires thrombopoietin, which is costly. Here, we aim to develop a more straightforward and affordable method. Synchronization of the MEG-01 cells was initially performed using serum-free culture, followed by spontaneous cell differentiation in the presence of serum. Different stages of megakaryoblast differentiation were classified based on cell morphology, DNA content, and cell cycle. The MEG-01 cells released platelet-like particles at a level comparable to that of the thrombopoietin-activated MEG-01 cells. The platelet-like particles were distinguishable from PLP-derived extracellular vesicles and could express P-selectin following ADP activation. Importantly, the platelet-like particles induced fibrin clotting in vitro using platelet-poor plasma. Therefore, this thrombopoietin-independent cell synchronization method is an effective and straightforward method for studying megakaryopoiesis and thrombopoiesis.

## Introduction

Platelets play a vital role in the homeostasis of the circulatory system by preventing bleeding and activating proper clot formation^1–3^. Impairment in platelet production leads to bleeding, a life-threatening condition^4^. Platelet transfusion is a standard practice needed for patients who have severe bleeding; however, this process depends on donor availability. Several studies have attempted to produce platelets in vitro from donor-independent sources^5,6^. Thus, understanding the mechanism of platelet generation requires an in vitro model. Platelets are produced from stem cells in bone marrow through two continuous processes: megakaryopoiesis and thrombopoiesis^7,8^. In megakaryopoiesis, CD34-positive hematopoietic stem cells differentiate into highly proliferative megakaryocyte/erythroid progenitors, which differentiate into three distinct stages of megakaryoblasts. Subsequently, megakaryocytes release platelets. Thrombopoietin (TPO) regulates thrombopoiesis but not megakaryopoiesis. Therefore, it is necessary to recapitulate these processes by deploying cell models and extrinsic regulators.

CD34-positive hematopoietic stem cells are useful for studying megakaryopoiesis and thrombopoiesis. Nevertheless, cell culture protocols remain complicated and expensive, limiting the large-scale production of platelets from CD34-positive hematopoietic stem cells^9–11^. Human cancerous cell lines are expandable in a less complicated culture system. The megakaryoblastic MEG-01 cell line is derived from leukemia patients and used as an in vitro model for studying megakaryopoiesis and thrombopoiesis. MEG-01 cells produce platelet-like particles (PLPs) when activated by TPO^12^. Since the TPO-based protocol is expensive, a simpler and lower-cost method to produce PLPs from MEG-01 cells independent of TPO might be useful. To develop such a protocol, the mechanisms of MEG-01 cell differentiation need to be elucidated. This study aims to examine the morphological changes that occur during the differentiation of MEG-01 cells and assess the production of functional PLPs. Through a growth retardation strategy, this study is the first to develop a more straightforward protocol for inducing PLP generation at an efficiency comparable to that of the TPO-based method. The critical success stems from the synchronization of MEG-01 cells to facilitate a homogenous cell stage that is then followed by the spontaneous production of PLPs.

## Results

### Synchronized MEG-01 cells exhibit morphological homogeneity and slow proliferation

**Figure 1:**
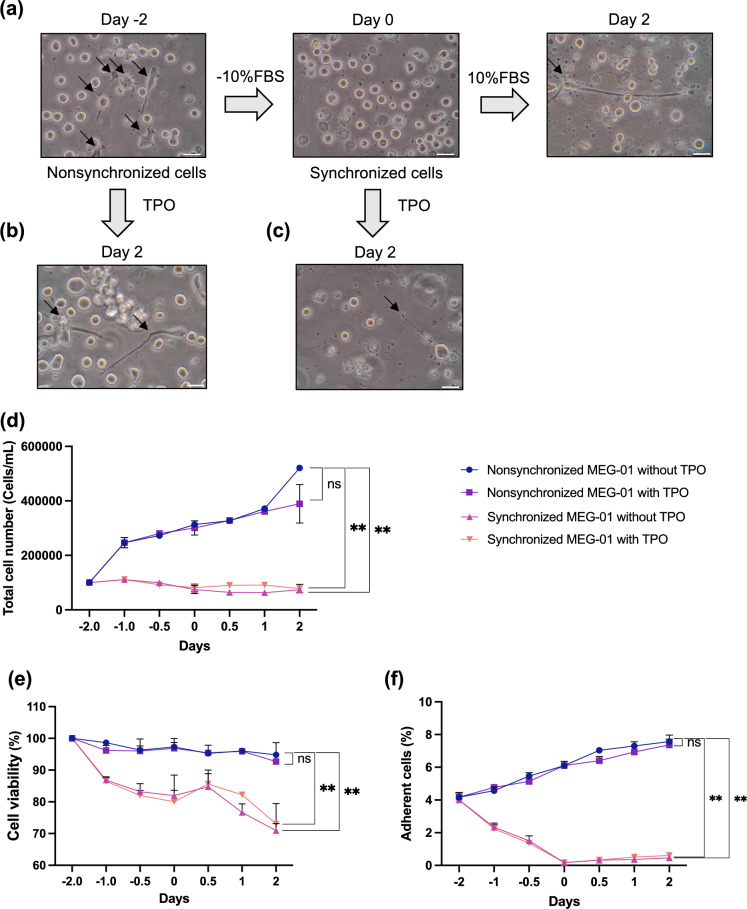
Morphology and proliferation of MEG-01 cells in asynchronous and synchronous culture. (**a**) Representative phase contrast images of MEG-01 cells under an inverted microscope. MEG-01 cells were cultured in FBS-supplemented culture medium (refer to day − 2 in the left panel). The cultured MEG-01 cells were subjected to synchronized culture for an additional 2 days (refer to day 0 in the middle panel). The synchronized MEG-01 cells were subsequently cultured in FBS-supplemented medium without TPO for 2 days (right panel). (**b**) The nonsynchronized MEG-01 cells were subsequently cultured in FBS-supplemented medium with TPO for 2 days (refer to day 2). (**c**) Synchronized MEG-01 cells were subsequently cultured in FBS-supplemented medium with TPO for 2 days (refer to day 2). The scale bar denotes 30 μm; 40 × objective. In all panels, arrows indicate cytoplasm-projecting adherent MEG-01 cells. (**d**) Total number of MEG-01 cells in synchronized and nonsynchronized cultures with or without TPO at different time points. (**e**) Viability of synchronized and nonsynchronized MEG-01 cells in the presence and absence of TPO at different time points. (**f**) Percentages of adherent MEG-01 cells under synchronized and nonsynchronized cultures with or without TPO at different time points. For (**b**–**d**), days − 2, − 1, and − 0.5 indicate synchronization and nonsynchronization phases, while days 0.5, 1, and 2 indicate periods post-TPO exposure. All experiments were repeated 3 times, and the data represent the mean ± standard deviation. Statistical analyses were performed using ANOVA. *ns* nonsignificant; **P < 0.01.

The stepwise protocol of cell synchronization and induction of cell differentiation is shown in Supplemental Fig. 1a. In the 2-day culture supplemented with 10% FBS, the morphology of the MEG-01 cells was heterogeneous, consisting of floating round cells and cytoplasm-protruding, adherent cells (arrows in left panel, Fig. 1a, Supplementary Fig. 1b). After removal of FBS from the culture media for 2 days, all cells were round without cytoplasm projection (middle panel, Fig. 1a, Supplementary Fig. 1c). Upon readding FBS into the cultures of the synchronized MEG-01 cells, we observed elongation of the cytoplasm (right panel, Fig. 1a, Supplementary Fig. 1d), similar to the conventional culture with TPO (Fig. 1b). However, the synchronized cells were not proliferative regardless of TPO exposure (Fig. 1c,d). The viability of the synchronized cells slightly decreased compared to that of the nonsynchronized cells in both the presence and absence of TPO (Fig. 1e). Moreover, the percentage of adherent cells in the FBS-deprived condition significantly decreased compared to that of the conventional culture (Fig. 1f). Thus, FBS deprivation resulted in morphological homogeneity, slow proliferation, and reduced adherence of viable MEG-01 cells.

### Synchronization of MEG-01 cells allows the classification of megakaryoblast stages

**Figure 2:**
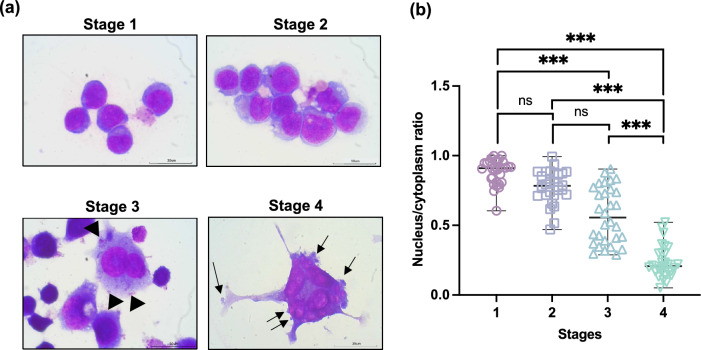
Classification of megakaryoblast stages of MEG-01 cells. The synchronized MEG-01 cells were classified based on the MGG-visualized morphology. (**a**) Representative images of MEG-01 cells at four different stages. Images were captured using a 100 × objective lens with oil fields. The scale bar denotes 30 μm. Arrowheads indicate azurophilic granules, while arrows indicate platelet-sized particles. (**b**) Scatter dot plot of the nucleus/cytoplasm ratio at each stage of MEG-01 cell differentiation. The sizes of the cell and nucleus were measured using the ImageJ program. Each dot represents individual cells (n = 30). *ns* nonsignificant; ***P < 0.001.

Synchronized MEG-01 cells were cultured for 2 days with FBS and classified into four stages of megakaryopoiesis. In stage 1, the cells had a basophilic cytoplasm and round shape (Fig. 2a, Supplementary Fig. 2a). Nuclei were round or oval without a lobe and located at the center or eccentric of the cells (Supplementary Fig. 2b, Table 1). In stage 2, the cells exhibited a round shape. Vacuoles were occasionally detected (Fig. 2a). The cytoplasm became less basophilic (Table 1). The nucleus was round or oval with 1–2 lobes and located at the center (Supplementary Fig. 2b, Table 1). Some cells had kidney-shaped nuclei. In stage 3, pseudopods and extensive blebbing membranes were observed (Fig. 2a). Fine azurophilic granules appeared in the cytoplasm that were pale blue with a pink cast (arrowheads; Fig. 2a). The cells became larger and varied in size (Supplementary Fig. 2a, Table 1). The nucleus remained round or oval and was located at the center (Fig. 2b, Supplementary Fig. 2b). One or more lobes of the nucleus were observed (Table 1). In stage 4, the cells had elongated pseudopods and cytoplasmic protrusions. Proplatelet-like structures and distinctive platelet-sized particles were clearly observed (arrows; Supplementary Fig. 1d,e and Fig. 2a). There were 1–12 lobes of the nucleus (Fig. 2a, Table 1). Thus, the synchronized MEG-01 cells undergo morphological changes similar to the four stages of megakaryoblast differentiation.

**Table 1:** Key characteristics of the MEG-01 cells classified into 4 different stages after the addition of serum when the cells were synchronized for 2 days.

Characterization	Stage 1	Stage 2	Stage 3	Stage 4
Morphology	Myeloblast-like cells	Promegakaryocyte-like cells	Megakaryocyte-like cells	Proplatelet-like cells
Cell size; µm Median (min–max)	18 (10–29)	25 (19–60)	42 (13–135)	81 (7–205)
N:C ratio	0.6–1:1	0.5–0.9:1	0.3–0.9:1	0.1–0.5:1
Nuclear shape	Round, oval	Round, oval, kidney shape	Round, oval, lobulated (2 or more lobes)	Round, oval, lobulated (2 or more lobes)
Nuclear position	Central or eccentric	Central or eccentric	Central	Central
Nuclear color	Red-purple fine	Red-purple, increase chromatin	Red-purple	Red-purple
Nucleoli	1–3	1–4	1–2	1–3
Nucleoli color	Purple	Purple	Purple	Purple
Membrane shape	Round	Round, occasional vacuole	Abundant pseudopodia, extensive membrane blebbing	Elongated pseudopod, cytoplasmic protrusions, proplatelet-like structures, and distinctive platelet-sized particles
Cytoplasmic color	Basophilic	Less basophilic	Pale blue with a pink cast	Pale blue with a pink cast
Number of lobes	0	1–2	1–8	1–12
Cytoplasmic granule	–	–	Fine azurophilic granules	Fine azurophilic granules

### Cytoplasmic protrusion of synchronized MEG-01 cells accompanied by F-actin polymerization during PLP production

**Figure 3:**
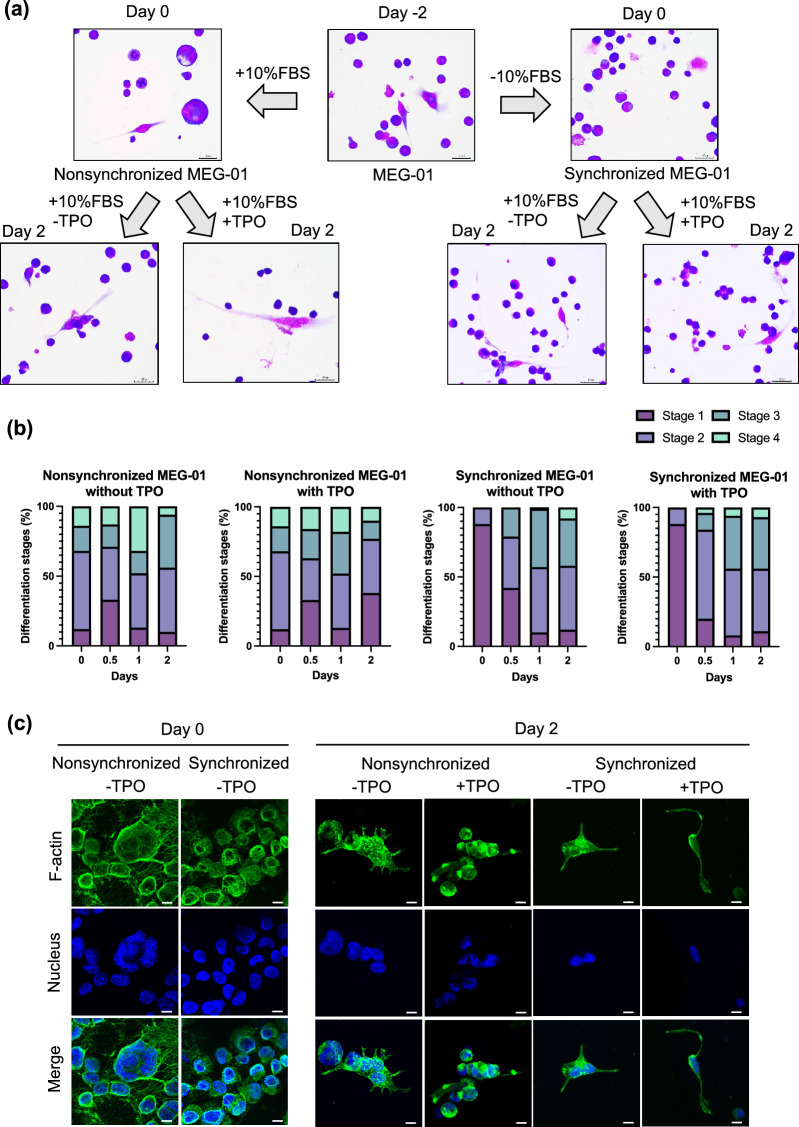
Morphological changes in nonsynchronized and synchronized MEG-01 cells during PLP production. (**a**) Representative microscopy images of MEG-01 cells adherent to a cover glass. MEG-01 cells were cultured in FBS-supplemented culture medium for 2 days (refer to day − 2 in the middle upper panel). The 2-day culture MEG-01 cells were subjected to nonsynchronized and synchronized culture for an additional 2 days (refer to day 0 in the left and right upper panel, respectively). The nonsynchronized or synchronized MEG-01 cells were subsequently cultured in FBS-supplemented medium with or without TPO for 2 days (lower panel). Images were captured using a 40 × objective lens. The scale bar denotes 30 μm. (**b**) Stacked bars show the proportion of MEG-01 cells in the four stages that adhered to the cover glass slides on days 0, 0.5, 1, and 2, with each day N = 100. (**c**) Confocal microscopy images of adherent cells labeled with anti-human F-actin (green). The nucleus was stained with 4,6-diamidino-2-phenylindole (DAPI, blue) on day 0 and day 2. Representative images were captured using a 40 × objective lens. The scale bar denotes 10 μm.

On day − 2 to day 0 of the nonsynchronous culture, the MEG-01 cells appeared in several sizes. Some cells were multinucleated (upper left panel Fig. 3a, day 0). After 2 days of nonsynchronous culture, the cells were subjected to 2 days of cell synchronization in the absence of FBS (upper right panel Fig. 3a, day 0). All cells were round and similar in size. No cells with cytoplasmic protrusions were observed. Then, the cells were grown in FBS-supplemented medium for an additional 2 days after cell synchronization (lower right panel Fig. 3a, day 2). The cells had polygonal shapes and cytoplasmic protrusions. On day 0, the synchronized cells were present only in stages one and two, but the nonsynchronized cells were present in all stages (Fig. 3b). On days 0.5 to 2, the proportion of stage three and four synchronized cells increased, and there was no difference between the nonsynchronized and synchronized cells (Fig. 3b). The pattern of cytoplasmic F-actin suggests cytoskeletal changes during thrombopoiesis^13^. Thus, we examined F-actin (Fig. 3c). Following an additional culture for 48 h without TPO, the nonsynchronized cells had spike-like cytoplasmic protrusions. Notably, the cytoplasmic protrusions of the synchronized cells were longer than those of the nonsynchronized cells (Fig. 3c, day 2). Therefore, the synchronized MEG-01 cells had polygonal shapes with F actin-based cytoplasmic protrusions during PLP production.

### Cell cycle progression of MEG-01 cells during synchronization and PLP formation

**Figure 4:**
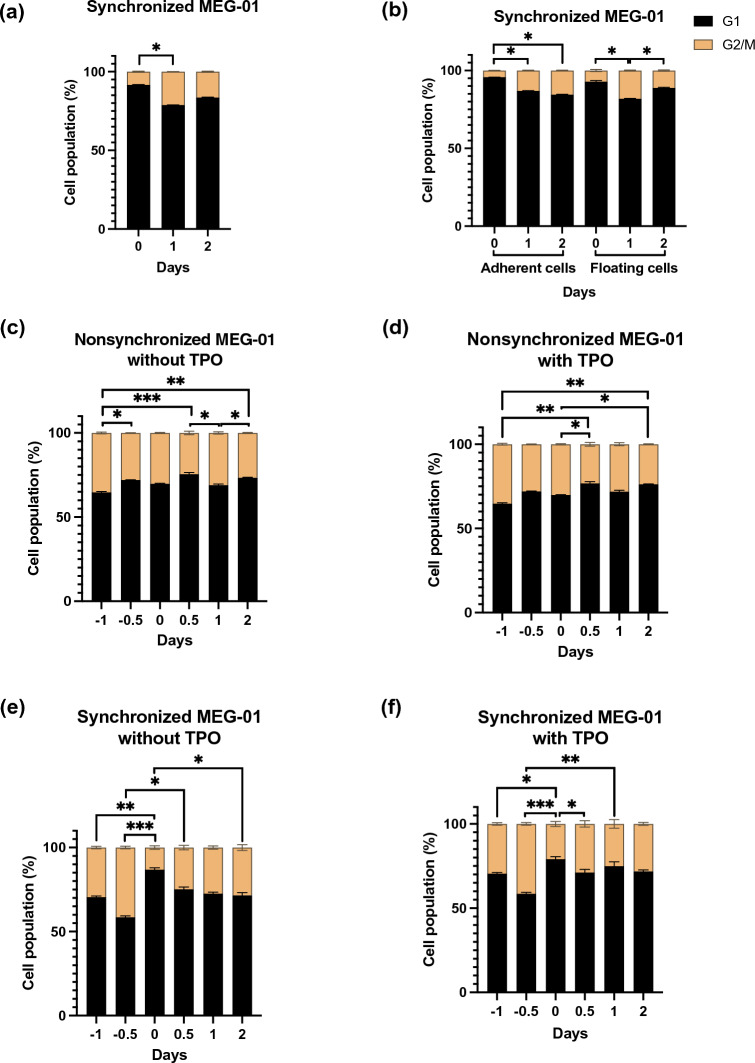
Cell cycle progression of MEG-01 cells during synchronization and PLP formation. (**a**) Stacked bars show the proportion of all synchronized MEG-01 cells in the G1 and G2/M phases at days 0, 1, and 2 during serum starvation. (**b**) Stacked bars show the proportion of adherent and floating synchronized MEG-01 cells in the G1 and G2/M phases at days 0, 1, and 2 during serum starvation. (**c**) Stacked bars show the proportion of nonsynchronized cells in G1 and G2/M at days − 1, − 0.5, 0, 0.5, 1, and 2 without TPO. (**d**) Stacked bars show the proportion of nonsynchronized cells in G1 and G2/M at days − 1, − 0.5, 0, 0.5, 1, and 2 with TPO. (**e**) Stacked bars show the proportion of synchronized cells in G1 and G2/M at days − 1, − 0.5, 0, 0.5, 1, and 2 without TPO. (**f**) Stacked bars show the proportion of nonsynchronized cells in G1 and G2/M at days − 1, − 0.5, 0, 0.5, 1, and 2 with TPO. All experiments were performed more than 3 times, and the data represent the mean ± standard deviation. *P < 0.05, **P < 0.01, ***P < 0.001.

Next, cell cycle progression was investigated after supplementing the culture of synchronized MEG-01 cells with FBS (Fig. 4a, Supplementary Fig. 3a). Both floating and adherent cells were subjected to cell cycle analysis (Fig. 4b, Supplementary Fig. 3b). On day 0 of culture, most of the synchronized cells were in the G1 phase (Fig. 4a). There was an increase in the proportion of synchronized adherent and floating cells in the G2 phase during the cell culture period (Fig. 4b). However, the percentage of cells in the cell cycle on day 2 was not different between the nonsynchronized and synchronized cells. Thus, it was unlikely that FBS supplementation accelerated cell cycle progression.

Since endomitosis occurs during megakaryopoiesis and multinuclear adherent MEG-01 cells appeared, as presented in Fig. 4b, the ploidy of the floating and adherent cells was analyzed separately. The percentage of the cell cycle was analyzed by examining gray zones in the histogram (Supplementary Fig. 3b). On day 0 before FBS addition, the adherent, synchronized MEG-01 cells had a low percentage of diploid cells. Following cell culture, the number of diploid cells increased on days 1 and 2. There was no difference in the percentage of diploid MEG-01 cells on day 2 between the synchronized and nonsynchronized cell cultures (Supplementary Fig. 3b).

For the cell cycle of the synchronized MEG-01 cells during PLP production, in a comparison of cell cycle differences between nonsynchronized cells with or without TPO, the proportion of G1 and G2 cells each day was observed. There was no difference (Fig. 4c,d). In contrast, compared to synchronized cells with or without TPO, the proportion of G1 cells increased and that of G2 cells decreased on day 0. After FBS addition, the proportions of G1 and G2 cells were similar to those of nonsynchronized cells (Fig. 4e,f). TPO is not effective for MEG-01 differentiation^14^, while the generation of platelet-like particles is independent of TPO^13,15,16^. Therefore, we examined the cell cycle of MEG-01 cells during exposure to valproic acid (VPA), a histone deacetylase inhibitor, which is able to induce MEG-01 cells to produce PLPs^13^. The percentages of G1 and G2/M cells in each condition did not differ significantly on days 7 and 14 postculture (Supplementary Fig. 3c–f). In summary, it was unlikely that FBS supplementation accelerated cell cycle progression and polyploidization.

### Synchronized MEG-01 cells release PLPs and PLP-derived extracellular vesicles in a TPO-independent manner

**Figure 5:**
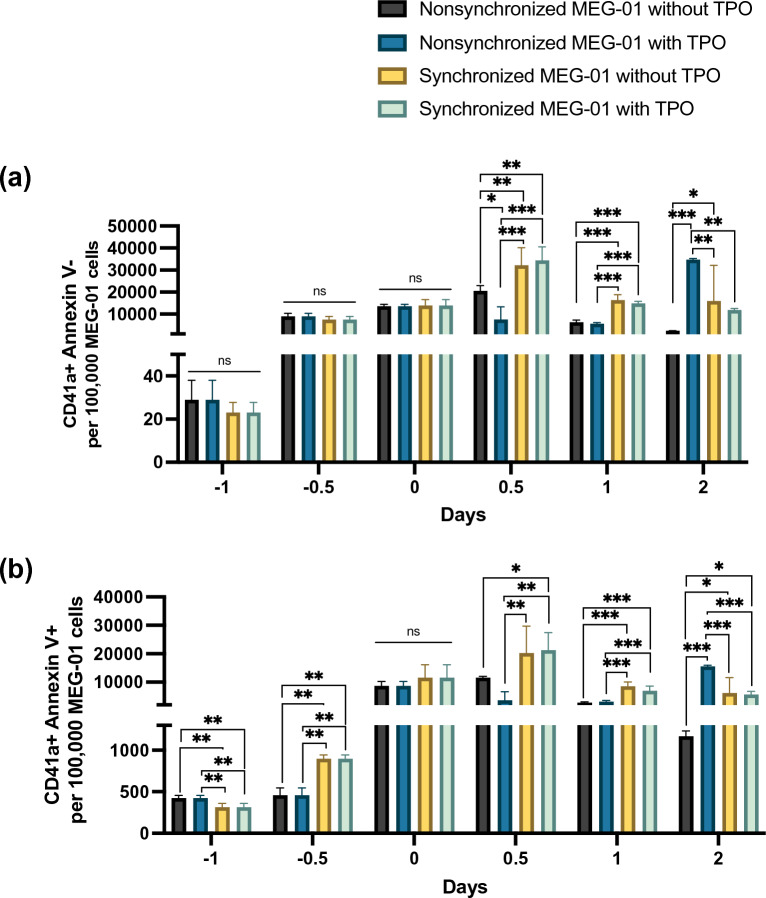
PLPs and PLP-derived EVs derived from synchronized MEG-01 cells. (**a**) The number of CD41a-positive Annexin V-negative PLPs released in the culture medium. (**b**) The number of CD41a-positive and Annexin V-positive EVs detected in the culture medium. On the Y-axis of both bar graphs, the total number of PLPs or EVs per 100,000 MEG-01 cells is displayed according to the culture period (days − 1, − 0.5, 0, 0.5, 1, and 2) on the X-axis. There were four different conditions: (1) nonsynchronized MEG-01 cells without TPO, (2) nonsynchronized MEG-01 cells with TPO, (3) synchronized MEG-01 cells without TPO, and (4) synchronized MEG-01 cells with TPO. All experiments were repeated 3 times, and the data represent the mean ± standard deviation. Statistical analyses were performed using ANOVA. *ns* nonsignificant; *P < 0.05, **P < 0.01, ***P < 0.001.

Based on the 1-µm beads, PLPs were larger than 1 µm, positive for CD41a and negative for phosphatidylserine on the surface, while PEVs were smaller than 1 µm and positive for CD41a and phosphatidylserine (Supplementary Fig. 4a). More than 30,000 PLPs were obtained from 100,000 synchronized MEG-01 cells cultured with or without TPO on day 0.5. Compared to that of the nonsynchronized cells treated with TPO, the total number of PLPs derived from the synchronized cells was 3 times higher on day 0.5 regardless of TPO exposure. Spontaneous release of PLPs from the nonsynchronized cells was the lowest among all examined conditions. In the presence of TPO, the total number of PLPs derived from the nonsynchronized cells was 3 times higher than that in the synchronized culture on day 2, suggesting a delayed production of PLPs in the nonsynchronized cells (Fig. 5a). The pattern of PEV release was similar to that of PLPs, although the number of PEVs tended to be lower than that of PLPs (Fig. 5b).

Next, we compared the total number of MEG-01-derived PLPs following exposure to VPA (Supplementary Fig. 4b,c). The nonsynchronized and synchronized cells produced the highest number of PLPs on day 7 (Supplementary Fig. 4b). However, the total number of PLPs derived from VPA exposure was lower than that derived from TPO induction. Notably, the mean fluorescence intensity of CD41a and CD42b was not significantly different among all culture conditions regardless of TPO, implying that PLP release is independent of TPO (Supplementary Fig. 4d–g). Overall, the synchronized MEG-01 cells spontaneously released PLPs and PEVs at levels higher and faster than the nonsynchronized MEG-01 cells that were exposed to TPO.

### The PLPs derived from synchronized MEG-01 cells can be activated

**Figure 6:**
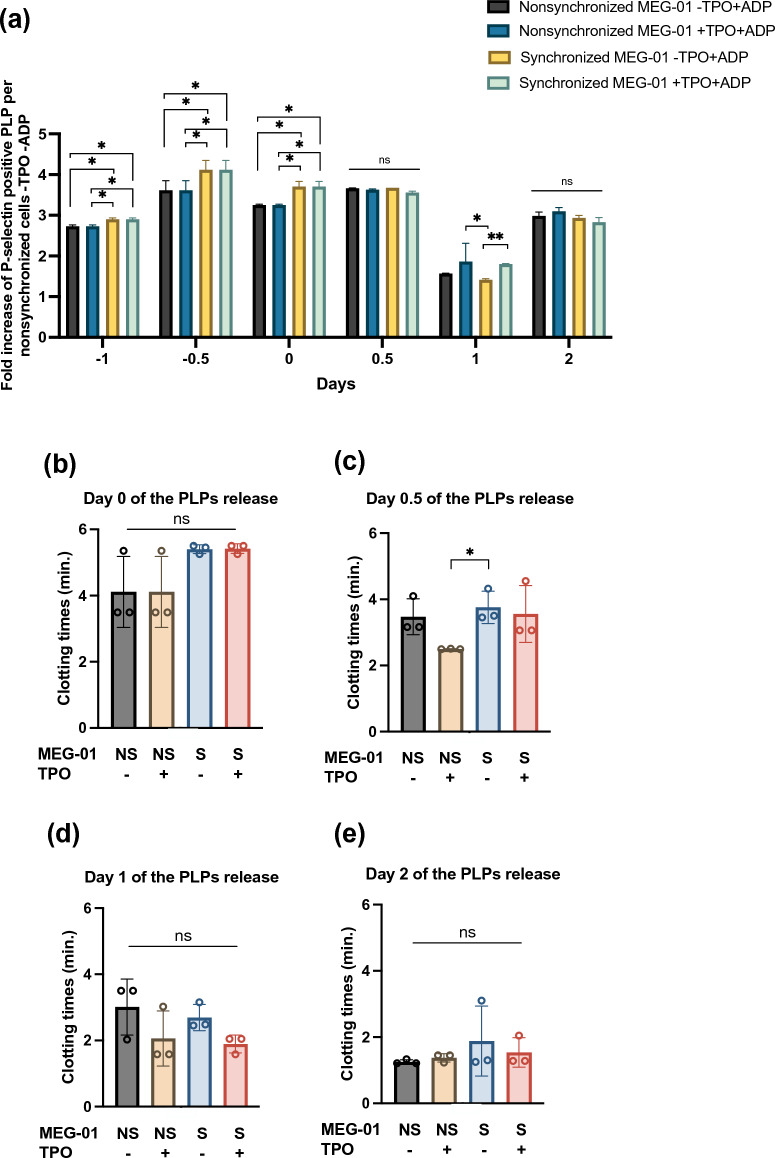
Functional assays of the PLPs derived from synchronized MEG-01 cells. (**a**) Fold increase in ADP-induced P-selectin expression on MEG-01-derived PLPs. The fold increase was calculated by dividing the percentage of P-selectin-positive PLPs obtained from all four conditions by that of the background signal. (**b**–**e**) Clotting time of platelet-poor plasma prepared by passing plasma through a 0.1-µm filter. For both panels, the PLPs were obtained from different culture experiments at days 0, 0.5, 1, and 2. There were (1) nonsynchronized MEG-01 cells without TPO, (2) nonsynchronized MEG-01 cells with TPO, (3) synchronized MEG-01 cells without TPO, and (4) synchronized MEG-01 cells with TPO. The data are from 3 independent experiments. *NS* nonsynchronized MEG-01 cells, *S* synchronized MEG-01 cells, *TPO* thrombopoietin, *ns* nonsignificance; *P < 0.05, **P < 0.01, ***P < 0.001.

ADP induced P-selectin expression on PLPs (Supplementary Fig. 5a). The percentage of P-selectin-positive PLPs released from the nonsynchronized cells that were not treated with TPO was set as the background control. Without ADP, 18.5% of the P-selectin-positive PLPs were derived from nonsynchronized cells. The fold increase in P-selectin-positive PLPs was calculated by dividing the percentage of P-selectin-positive PLPs by the baseline value of 18.5%. There was no difference in the fold increase in P-selectin among the experiments on days 0.5 and 2 post-TPO induction, but it decreased on day 1 (Fig. 6a). Compared to that of the PLPs derived from the nonsynchronized cells, the relative increase in ADP-induced P-selectin expression was different in the PLPs derived from the synchronized cells with or without TPO induction on days − 1, − 0.5, and 0 (Supplementary Fig. 5b–d). After the addition of FBS, the relative increase in ADP-induced P-selectin expression was not different in the PLPs derived from synchronized MEG-01 cells with or without TPO induction on days 0.5, 1, and 2 (Supplementary Fig. 5e–g). Notably, adding TPO into the culture of synchronized MEG-01 cells resulted in the release of PLPs that had no effect on P-selectin expression following ADP activation. Thus, synchronized MEG-01 cells spontaneously release functional PLPs independent of TPO.

### The PLPs derived from synchronized MEG-01 cells activate fibrin clots

Fibrin clots form faster in PRP than in PPP (Supplementary Fig. 5h). The time needed for the PPP to form a fibrin clot indicates the function of the MEG-01-derived PLPs. A shorter fibrin clot time implies a greater proportion of functional platelets. In all culture conditions of the MEG-01 cells, fibrin clotting time decreased over the period of PLP induction culture (Supplementary Fig. 5i–l). The PLPs derived from day 0 to 2 postculture of the synchronized cells without TPO exhibited no difference in the time of fibrin clot formation compared to the PLPs obtained from the TPO-induced culture of the synchronized cells (Fig. 6b–e). Notably, adding TPO to synchronized cells did not affect the proportion of PLPs capable of activating fibrin clots. These data suggest that the PLPs released from synchronized MEG-01 cells without TPO induction could activate fibrin clots.

### Substantial changes in ROS and apoptosis occur during cell synchronization and PLP formation

**Figure 7:**
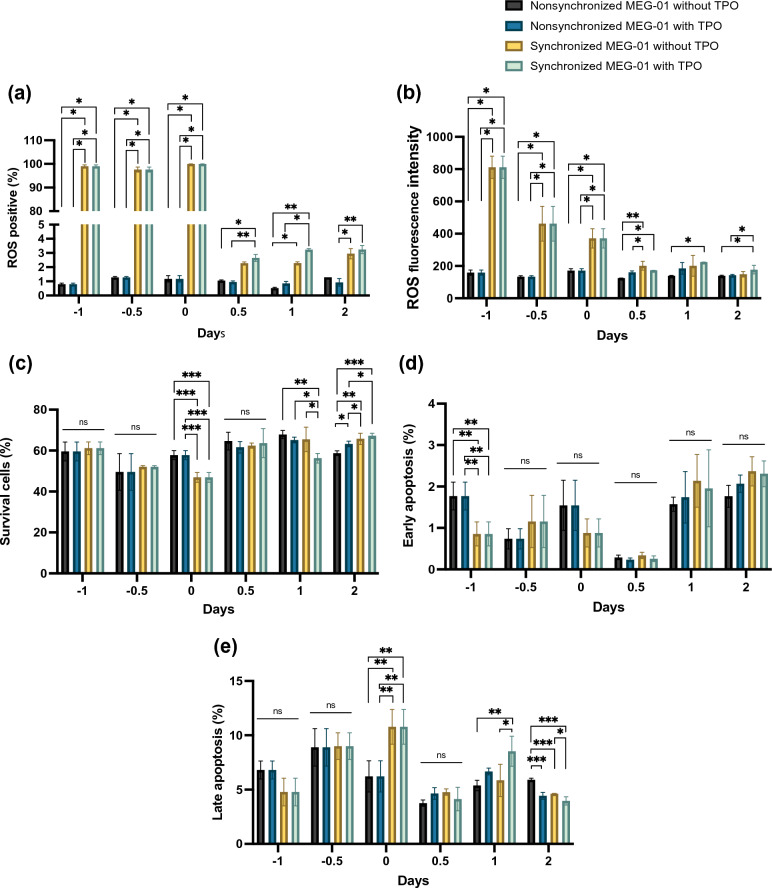
Substantial changes in ROS and apoptosis in MEG-01 cells during synchronization and PLP formation. The bar graph shows (**a**) the percentage of ROS-positive cells and (**b**) the ROS fluorescence intensity under various conditions at days − 1, − 0.5, 0, 0.5, 1, and 2. The bar graph shows (**c**) the percentage of surviving cells, (**d**) early apoptosis, and (**e**) late apoptosis at days − 1, − 0.5, 0, 0.5, 1, and 2. *ns* nonsignificant; *P < 0.05, **P < 0.01, ***P < 0.001.

High amounts of ROS are produced under nutritional oxidative stress^17^. ROS affect platelet generation and function^14,18^. In synchronous cell culture, almost 100% of cells were positive for ROS after withdrawal of FBS (days − 1, − 0.5 and 0 in Supplementary Fig. 6a). Following the addition of FBS to the synchronized cells, the percentages of ROS-positive cells gradually declined (days 0.5, 1, and 2 in Supplemental Fig. 6a and Fig. 7a). Similar to the percentage, the fluorescence intensity of ROS decreased (Fig. 7b, Supplementary Fig. 6b). ROS in nonsynchronized cells were present to some degree (2%), increasing to approximately 58% of the mean intensity of whole images after synchronized MEG-01. Hence, FBS deprivation-based cell synchronization increased ROS levels.

Compared to those of the nonsynchronized culture, the percentages of surviving cells slightly decreased after 24 h post FBS starvation (day 0; Fig. 7c). There was no significant increase in the number of early and late apoptotic cells (Fig. 7d,e). After FBS was added to the synchronized cells, the percentage of viable cells returned to levels comparable to those of the nonsynchronized cells on days 0.5, 1, and 2 (Fig. 7c). There were no changes in the percentages of early apoptotic cells after FBS resumption in any of the experiments (Fig. 7d). Notably, a significant increase in the number of late apoptotic cells was observed after adding TPO into the synchronized culture for 24 h (day 1; Fig. 7e). The decline and resumption of cell survival were independent of TPO. In contrast, VPA exposure significantly reduced cell viability by more than twofold (Supplementary Fig. 6c). This trend was consistent with the increase in early and late apoptotic cells (Supplementary Fig. 6d,e). Therefore, cell synchronization and platelet formation did not affect cell survival.

## Discussion

To our knowledge, serum starvation-based synchronization of megakaryoblastic MEG-01 cells is a novel method that allows megakaryopoiesis and platelet production in a TPO-independent manner. This method yielded quantity and quality of platelet-like particles at levels similar to the conventional method, in which nonsynchronized MEG-01 cells are exposed to TPO. Culture of MEG-01 cells is simpler than that of megakaryoblastic CMK and UT-7/TPO cells. The growth and proliferation of UT-7/TPO cells require TPO^19^, while those of CMK cells rely on the stimulation of interleukin-3 and granulocyte–macrophage colony-stimulating factor^20^. In contrast, FBS is sufficient for the growth and proliferation of MEG-01 cells. MEG-01 cells are derived from the bone marrow of a patient with chronic myelogenous leukemia carrying the Philadelphia chromosome and positive for glycoprotein (GP) IIb/IIIa antigen (CD41). Despite the presence of floating cells, half of the cultured MEG-01 cells adhere to plastic surfaces and protrude pseudopods^21^. MEG-01 cells are reportedly capable of producing PLPs without stimulation, albeit with low efficiency. The use of exogenous stimuli induces MEG-01 cells to undergo differentiation into mature megakaryocytes and release PLPs. These stimuli are phorbol diesters^22^, plant extracts with tumor-promoting activity^23^, valproic acid^13,15^ and TPO^24,25^. Moreover, the MEG-01 cell line has been used as an in vitro model for elucidating the mechanism underlying dengue virus-mediated thrombocytopenia^16^ and thrombin-activated platelets^26^. Despite its utility, the cell morphology and cell cycle during the differentiation of MEG-01 cells remain uncharacterized owing to the heterogeneity of the cell population. With the execution of cell synchronization, it is feasible to use May–Giemsa staining to classify the differentiation of MEG-01 cells into four distinct stages. However, cytochemistry techniques such as myeloperoxidase, alkaline phosphatase, acid phosphatase, alpha-naphthyl acetate esterase, or alpha-naphthyl butyrate esterase may allow for more detailed classification.

A conventional method for stimulating MEG-01 cells to undergo differentiation and platelet generation relies on commercial TPO. As an alternative to commercial TPO, previous studies demonstrated the use of genetically engineered cells for producing TPO. The GP + E-86 packaging cell line carries a gene encoding murine TPO^27^. Transplantation of this cell line into mouse bone marrow led to significantly elevated TPO levels in plasma (10^4^ U/mL)^28^. Thus, it is feasible to produce TPO in a lab by purifying TPO from the culture medium. Notably, the use of cell line-derived TPO is cost-saving compared to commercially purchased TPO. Nevertheless, the use of the GP + E-86 cell line requires additional time for TPO preparation before initiating MEG-01 differentiation into platelets.

Analysis of MEG-01 cell-based megakaryopoiesis is limited by the heterogeneity of megakaryoblasts in differentiation stages. Synchronized fourth-stage MEG-01 cells demonstrated the release of functional PLPs in a thrombopoietin-independent manner. Due to their leukemia origin, MEG-01 cells grow exponentially in a nonsynchronous manner. Analysis of cell differentiation is indeterminate as a result of the heterogeneity of the cell cycle stage. It was difficult to classify megakaryoblast stages in the heterogeneous population. Thus, a way to prepare the cells at the same stage allows more accurate cell analysis. There are several methods for cell synchronization based on the inhibition of cell cycle progression by using chemicals or the inhibition of cell metabolism by deprivation of amino acids^29^. Here, deprivation of FBS in routine culture resulted in a homogenous population of MEG-01 cells. Compared to the nonsynchronized MEG-01 cells, the synchronized MEG-01 cells exhibited 4 sequential stages of differentiation. Insufficient nutrition results in imbalances in pro-oxidant and antioxidant loads, leading to oxidative stress^17^. Notably, ROS reportedly mediate megakaryopoiesis and platelet generation from human bone marrow^14,18^. The osteoblastic niche is where MK differentiation from HSCs takes place, which is partly explained by the strong association between ROS and MKs^30^. Thus, it is likely that ROS may be involved in megakaryopoiesis of synchronized MEG-01 cells.

The in vitro culture of MEG-01 cells contained cell debris and PEVs in addition to PLPs. Cell debris could be identified based on the loss of membrane integrity. Moreover, PEVs express CD41a, a marker of platelets^31^. Hence, the use of anti-CD41a was insufficient to distinguish platelets from PEVs. Here, we used 1-µm beads as a cutoff to exclude PEVs, which were smaller than the beads. Assessment of thrombopoiesis was more accurate when using beads and antibodies. Moreover, the functions of mature platelets could be examined using several in vitro assays, including platelet aggregation, platelet activation, and thrombin activation. The purification of platelets without minimal activation is difficult, and platelet aggregation and activation are affected by PEVs in the cell culture medium. Although we provided a way to analyze the differentiation of MEG-01 cells and PLPs, the utility of this proposed method needs to be validated further.

In comparison to induced pluripotent stem cells (iPSCs), using MEG-01 cells along with cell synchronization offers distinct advantages. The method to generate platelets from human iPS cells is well established and produces more platelets than MEG-01 cells. Considering the technical perspective, there are drawbacks in the use of human iPS cell-based platelet production. First, this process is resource intensive. Human iPS cells grow on matrix protein-coated surfaces with stem cell culture medium and pluripotency-controlling growth factors^32^. As a result, culture of human iPS cells is more expensive than that of human cancer cell lines. Moreover, the differentiation of human iPS cells into platelets requires several types of cytokines and growth factors at each step and consumes a longer time compared to a single step of synchronization of the MEG-01 cells. In addition to simplicity and cost effectiveness, an advantage in the use of the MEG-01 cell line is scalability. Since the MEG-01 cells are highly proliferative using the conventional medium supplemented with only FBS, the starting number of the MEG-01 cells is scaled up. Overall, the MEG-01 cell line offers a simpler cultivation process, lower cost, and quicker platelet generation.

Nevertheless, cell synchronization decreases cell viability and cell adhesion and does not have a strong beneficial effect on the parameters tested compared to nonsynchronization. Cell synchronization provides a homogenous population, allowing accurate analysis of the output parameters resulting from cell differentiation. Biologically, the synchronizing MEG-01 cells had higher ROS production than nonsynchronized MEG-01 cells. ROS reportedly enhance the efficiency of platelet production, theoretically contributing to thrombopoiesis and platelet production^18,30,33^. Thus, cell synchronization allows the study of ROS in megakaryopoiesis and thrombopoiesis. From a technical perspective, FBS removal is simple and involves a straightforward technique for synchronizing MEG-01 cells. This approach is convenient and less complex than other synchronization methods that may involve intricate techniques such as chemical inhibitors, gene manipulations, or specialized equipment. While synchronized MEG-01 cells may encounter challenges related to cell viability and cell adhesion, a significantly different result was obtained. After reintroducing FBS, the synchronized MEG-01 cells produced a higher percentage of CD41a-positive Annexin V-negative PLPs than nonsynchronized MEG-01 cells (Fig. 5). Hence, these benefits of cell synchronization outweigh the abovementioned drawbacks regarding cell viability.

Considering the translation of our findings in the context of pathophysiology, TPO is essential for megakaryopoiesis and thrombopoiesis. In addition, some studies suggest the existence of TPO-independent megakaryopoiesis. Under normal hematopoiesis, mice lacking TPO and its receptor (c-mpl) have sufficient platelets^34^. Human subjects who lose functional c-Mpl still have a normal range of platelets^35^. Under physiological stress, platelets increase transiently during inflammation^36^. Proinflammatory IL-1α reportedly acts on mature megakaryocytes, resulting in cell rupture and subsequent proplatelet shedding in mouse bone marrow^37^. Administration of insulin-like growth factor (IGF)-1 in lethally irradiated, c-mpl-knockout mice increases platelets^38^. The chemokine CCL5 also upregulated proplatelet generation from in vitro culture of mouse megakaryocytes^39^. Given that IL-1α, IGF-1 and CCL5 often increase during inflammation, it implies a need for platelets under such physiological stress. Whether these cytokines regulate megakaryopoiesis under noninflammatory conditions and in a TPO-independent manner remains elusive^40^. Thus, a method for the study of TPO-independent platelet production is essential and could be demonstrated using our method.

## Materials and methods

### Maintenance of the MEG-01 cell line

The MEG-01 cell line (ATCC, MD) was cultured in RPMI-1640 medium with 10% heat-inactivated fetal bovine serum (FBS, HyClone, US), hereafter called RPMI/FBS. Cells were incubated at 37 °C with 5% CO_2_. Before synchronization, cells were cultured in RPMI/FBS for 2–3 passages. For subculture, cells were collected using a 13-mm cell scraper (BLADE, SPL Life Sciences, Korea). To observe the morphology of adherent cells, a 12-mm^2^ round glass coverslip was placed in a 24-well plate, followed by seeding cells at a concentration of 1 × 10^5^ cells/mL.

### Generation and isolation of PLPs

Cells were exposed to 100 ng/mL recombinant human TPO (PeproTech, NJ) or 2 mM valproic acid (Sigma-Aldrich, St Louis) in RPMI/FBS at 37 °C with 5% CO_2_ for 48 h^13,15,24^. To isolate PLPs, the culture supernatant was centrifuged at 161×*g* for 5 min at RT.

### Synchronization of the MEG-01 cell line

Cells were cultured without FBS for 2 days (refer to day − 2 as 2 days before the readdition of FBS). Then, the cells were collected, washed with PBS, and cultured in RPMI/FBS for an additional 2 days (referred to as day 2, a period after the readdition of FBS). To induce megakaryopoiesis and thrombopoiesis, the cells were cultured in RPMI/FBS with 100 ng/mL human TPO at a density of 1 × 10^5^ cells/mL and incubated at 37 °C with 5% CO_2_ for 48 h. Cell viability was examined using 0.4% trypan blue and a hemocytometer.

### Classification of the MEG-01 cell line

Cells were cultured on a glass coverslip and stained with May–Grunwald–Giemsa (MGG). A total of 200 cells were examined and classified according to the megakaryoblast stage^41^ under a light microscope (Olympus CX31). Images were captured and edited with the KoPa capture program.

### Actin polymerization

For visualization of actin polymerization in the protruding cytoplasm, cells were prepared as adherent cells on a glass coverslip. After washing with PBS, the cells were incubated with 1% paraformaldehyde in PBS at RT for 30 min. The cell membrane was permeabilized using 0.05% Triton X-100 in PBS at 20–25 °C for 15 min. After washing with PBS and incubation with 1% BSA in PBS, the cells were incubated with a mouse monoclonal anti-F-actin antibody (1:100, Abcam) at 4 °C overnight, washed with PBS and incubated with Alexa Fluor 488-conjugated goat anti-mouse IgG (1:100, Invitrogen) at 20–25 °C for 30 min. A drop of Fluoroshield Mounting Medium with DAPI (Abcam, UK) was added. The cells were visualized under a confocal microscope (Nikon A1R, Nikon Corporation).

### Cell cycle analysis

The BD Cycletest™ Plus DNA Reagent Kit (BD Biosciences, CA) was used following the manufacturer's instructions. Briefly, cells were fixed with 70% prechilled ethanol at 4 °C overnight. The cells were incubated with trypsin buffer followed by the addition of trypsin inhibitor and RNaseA. The cells were then stained in propidium iodide and incubated at 2–8 °C for 30 min with light protection. After washing with PBS, the cells were analyzed using a BD FACSCalibur flow cytometer (BD Bioscience).

### Flow cytometry of PLPs and PEVs

The culture supernatant was collected and centrifuged at 161×*g* for 10 min at RT. The supernatant was mixed with PE-conjugated anti-human CD41a antibody (BD Pharmingen, CA), APC-conjugated anti-human CD42b antibody (ImmunoTools, Germany) and FITC-conjugated annexin V (Miltenyi Biotec, CA). As a positive control, venous blood was collected in a 3.2% sodium citrate tube followed by centrifugation at 161×*g* for 10 min to prepare platelet-rich plasma (PRP). Cells were analyzed using a FACSCalibur flow cytometer. One-micron beads (Spherotech, IL) were set as a size separator between PLPs and PEVs. Data were analyzed using FlowJo software (Treestar, OR).

### PLP activation test

PLPs were stimulated using 0.2 mM adenosine diphosphate (ADP) (Sigma-Aldrich, Singapore) at 37 °C for 15 min and fixed with an equal volume of 0.1% paraformaldehyde (Sigma-Aldrich, Singapore) for an hour. The fixed PLPs were incubated with 5 µL of APC-conjugated anti-human CD62P antibodies (Biolegend, San Diego, CA) and subjected to analysis using a FACSCalibur flow cytometer.

### Plasma clotting time

Platelet-poor plasma (PPP) was prepared by passing plasma through a 0.1-µm filter. PPP was incubated with 25 mM CaCl_2_ in a glass tube at 37 °C with gentle shaking. Plasma clotting time was measured as the time it took the plasma to become a fibrin-strand-like gel. The endpoint of the plasma clotting time was when there was no movement in response to shaking.

### Reactive oxygen species (ROS) assay

ROS production was measured using a 2′,7′-dichlorofluorescein diacetate (DCFDA) assay kit (Abcam, MA) according to the manufacturer’s instructions. The cell suspension was incubated with DCFDA for 30 min with light protection. Fluorescence intensity was examined using a BD FACSCalibur flow cytometer (BD Bioscience, CA). Mean fluorescence intensity was measured using ROI statistics from the NIS-Elements AR Nikon program.

### Cell apoptosis assay

Cell apoptosis was detected using the Annexin V-FITC Apoptosis Detection Kit (Miltenyi Biotec, CA). After washing once with PBS, the cells were incubated with 5 µL of Annexin V-FITC and 5 µL of propidium iodide for 30 min with light protection. Cells were analyzed using a BD FACSCalibur flow cytometer (BD Bioscience, CA).

### Statistics

The results are presented as the mean ± standard deviation (SD) of the mean, with *n* representing the number of biological replicates unless otherwise stated. Statistical *P* values were calculated by analysis of variance (ANOVA), followed by Bonferroni post hoc comparisons between individual experimental groups (SPSS SigmaStat 28.0.0.0 software). All other data were analyzed by GraphPad (Prism 9.0). Significance levels are noted with * for *P* < 0.05, ** for* P* < 0.01, and *** for *P* < 0.001.

### Ethics statement

This study was performed after obtaining approval from the Ethical Review Committee for Research Involving Human Subjects in Research, Chulalongkorn University, in accordance with the International Conference on Harmonization-Good Clinical Practice, which is guided by local policy, national law, and the World Health Association Declaration of Helsinki (COA No. 159/66).

## Conclusions

Serum starvation-induced synchronization of MEG-01 cells enables spontaneous PLP generation without TPO. Oxidative stress likely influences the megakaryopoiesis of synchronized MEG-01 cells; moreover, the fourth stage of synchronized MEG-01 cells release functional PLPs independent of TPO. Although our methodology in PLP generation is promising, further validation is needed to assess its effectiveness in comparison with other methods using different cell types and stimuli.

### Supplementary Information

Supplementary Figures.

## Data Availability

The datasets used and/or analyzed during the current study are included within the manuscript, and the details are uploaded as supplementary files.
