# Supplementary material for: Thrombopoietin-independent generation of platelet-like particles from megakaryoblastic cells
Source: Sci Rep. 2023 Dec 18;13:22553. doi: 10.1038/s41598-023-50111-6 (PMC10728061; doi:10.1038/s41598-023-50111-6)
Supplement: Supplementary file 1 — Supplementary Figures. [file 41598_2023_50111_MOESM1_ESM.pdf]

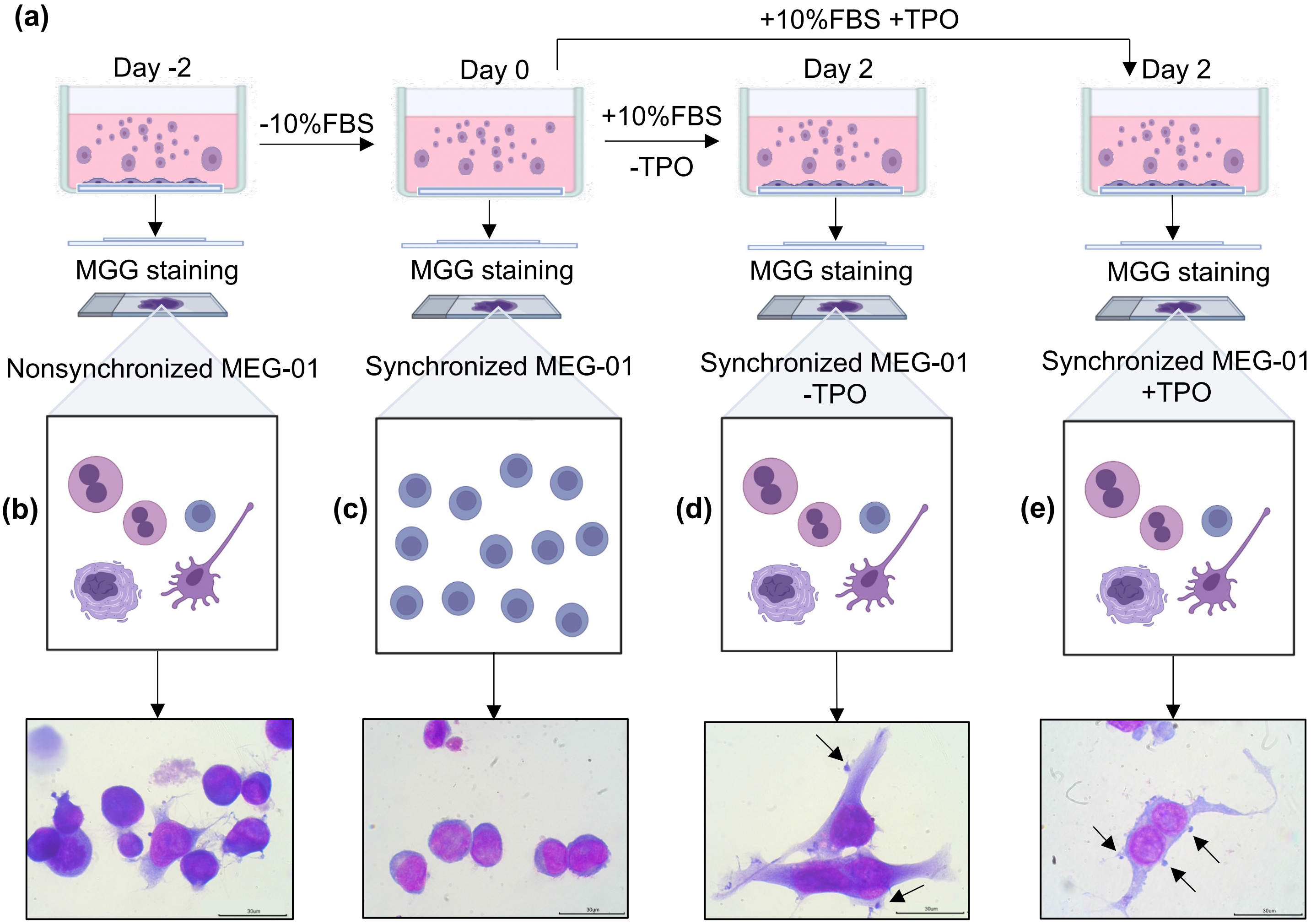

Supplementary Figure 1. Morphological changes and proliferation of TPO-independent synchronized MEG-01 cell culture during PLP production. (a) Schematic diagram of nonsynchronized and synchronized cells, PLP formation and cell morphological analysis at different time points. MGG staining was used to stain cells that were adherent to cover glass. (b) MEG-01 cells were cultured in RPMI/FBS to produce a nonsynchronized culture (presented as a mixture of cells with different sizes and morphology). Representative images of MGG-stained day -2 nonsynchronized MEG-01 cells cultured in RPMI/FBS. (c) For synchronization, MEG-01 cells were cultured in the same medium without FBS for 2 days. The synchronized cells are presented as dark blue circles of similar size. Representative images of MGG-stained MEG-01 cells synchronized for 2 days in RPMI only (day 0). (d) Synchronized MEG-01 cells were cultured without TPO to examine the spontaneous generation of PLPs. PLP generation was set for 48 hours of culture. Representative images of MGG-stained MEG-01 cells synchronized for 2 days followed by culture in RPMI/FBS without TPO for an additional 2 days. (e) Synchronized MEG-01 cells were cultured in FBS-supplemented medium with TPO to induce PLP production. Representative microscopy images of day 2 synchronized MEG-01 cells cultured in TPO-supplemented RPMI/FBS for an additional 2 days. Images were captured using a 100x objective lens with oil fields. The scale bar denotes 30 μm; 100× objective. Arrows indicate the platelet-sized particles.

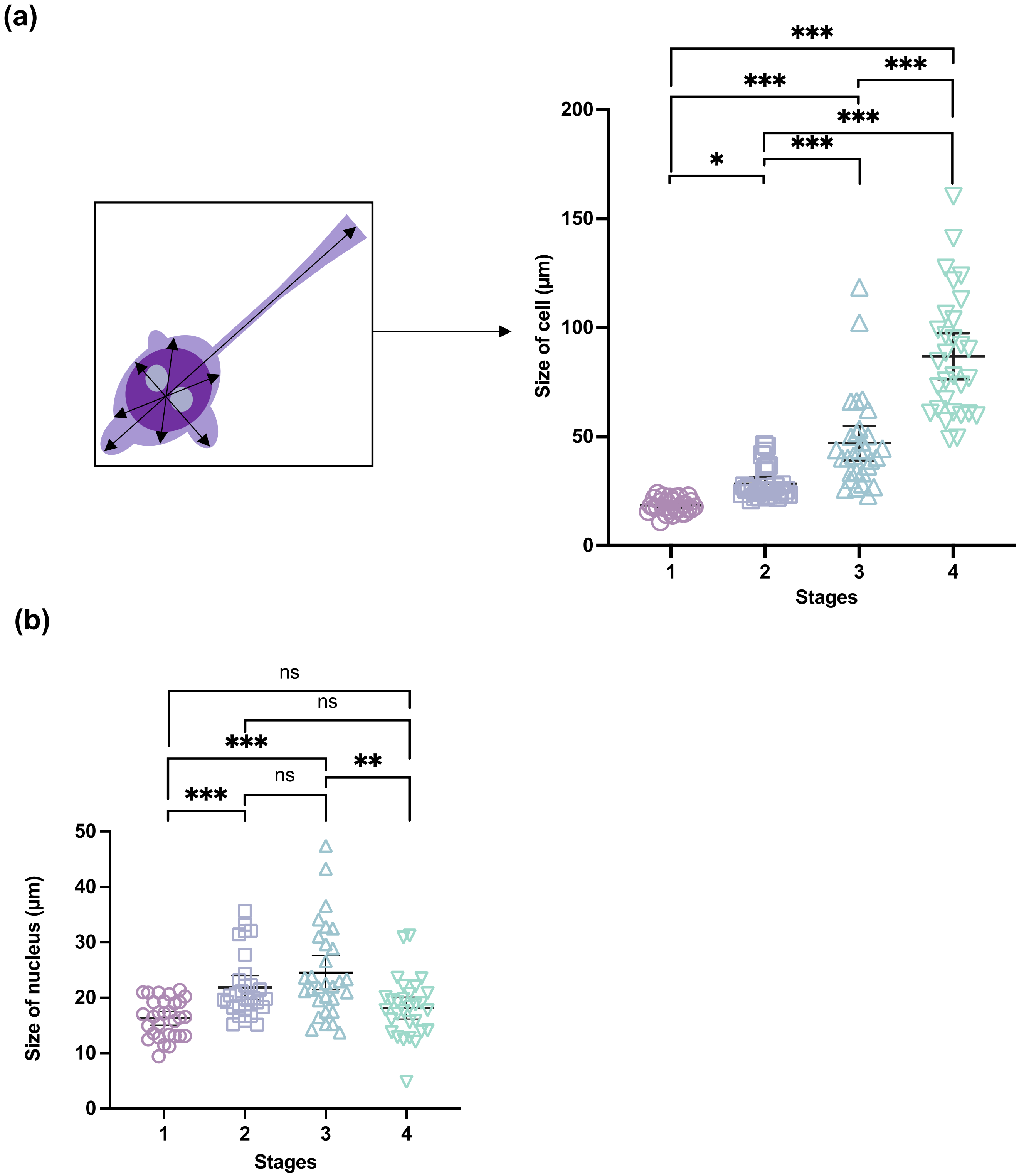

Supplementary Figure 2. Classification of differentiation stages of MEG-01 cells. Synchronized MEG-01 cells were classified based on morphology illustrated using MGG staining. (a) Scatter dot plot of cell size (μm) at each stage. (b) Scatter dot plot of the nucleus size (μm) at each stage. The sizes of the cell and nucleus were measured using the ImageJ program, with each stage N=30. Statistical analyses were performed using ANOVA. ns, nonsignificance; *P <.05, **P <.01, ***P <.001.

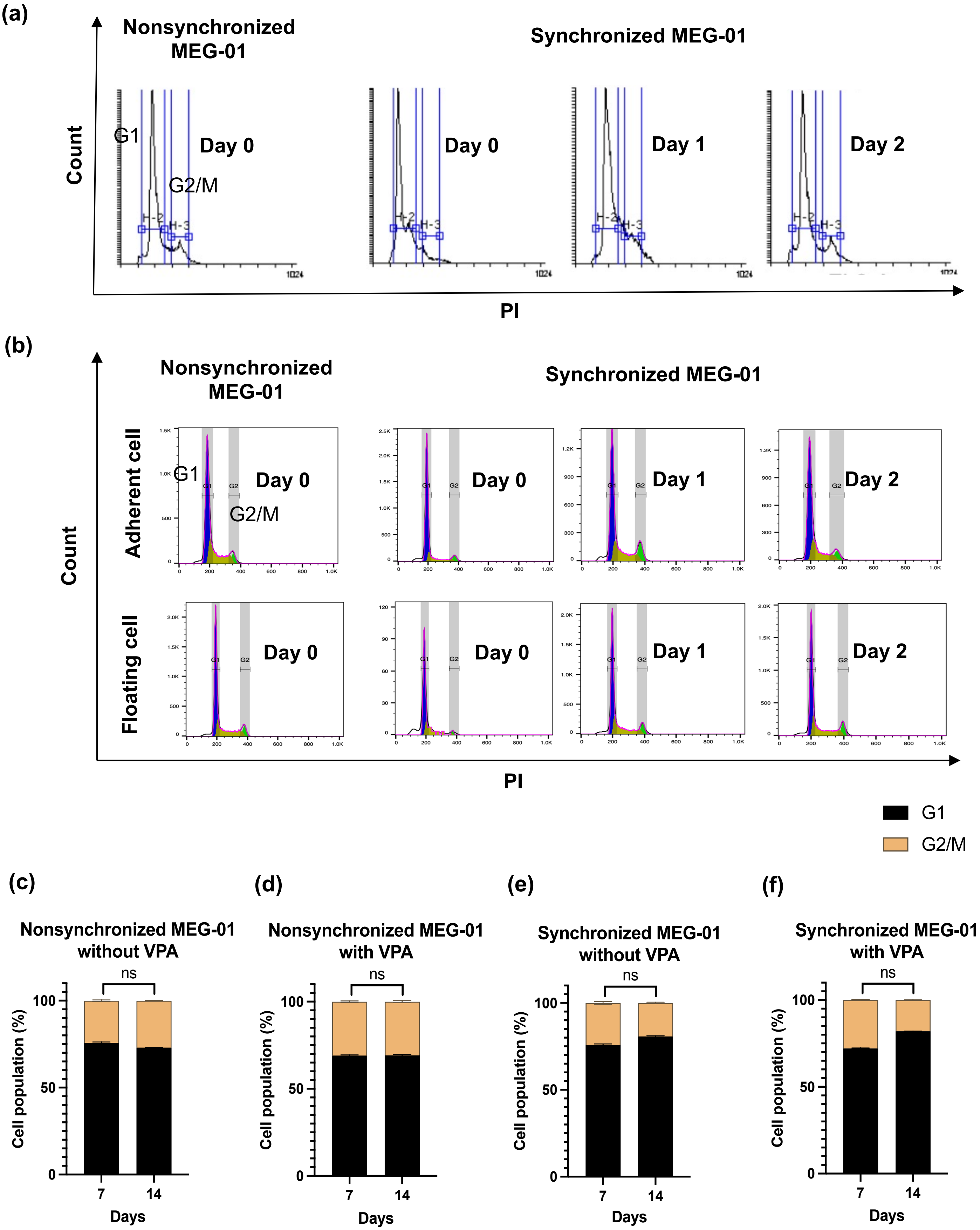

Supplementary Figure 3. Cell cycle of MEG-01 cells during PLP formation. (a) Histograms showing the cell cycle of the nonsynchronized and synchronized MEG-01 cells at days 0, 1 and 2 without TPO. DNA in the nucleus was labeled using propidium iodide (PI). DNA content was used to determine the cell cycle stage levels. (b) Histograms showing cell cycle levels (gray zones) of the floating and adherent MEG-01 cells. (c) The proportion of nonsynchronized cells in G1 and G2/M at days 7 and 14 without or (d) with VPA. (e) The proportion of synchronized cells in G1 and G2/M at days 7 and 14 without or (f) with VPA. ns, nonsignificance.

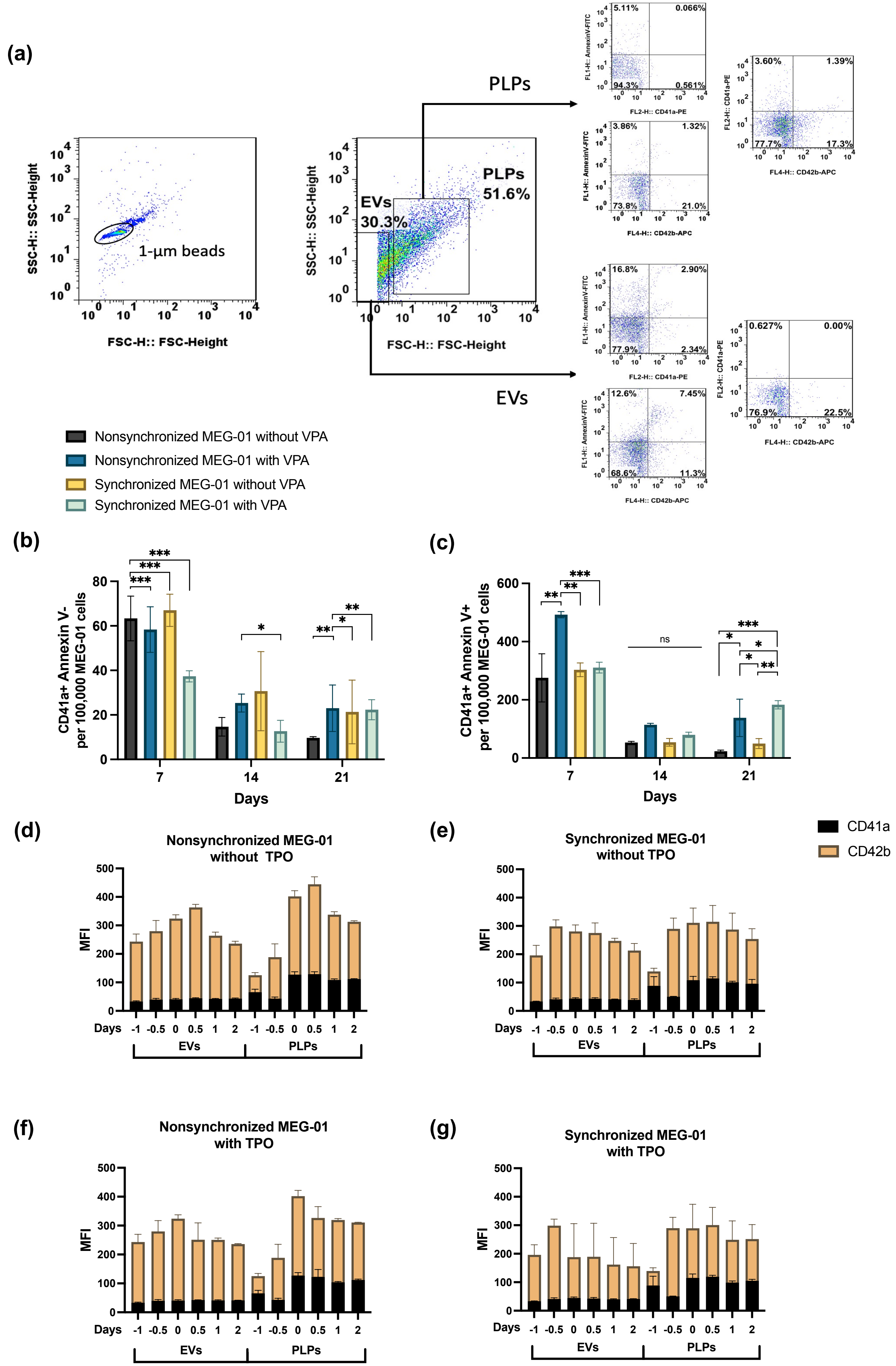

(a)
1-μm beads
SSC-H:: SSC-Height
FSC-H:: FSC-Height
EVs
30.3%
PLPs
51.6%
PLPs
EVs
5.11%
0.066%
94.3%
0.561%
FL1-H:: AnnexinV-FITC
FL2-H:: CD41a-PE
3.86%
1.32%
73.8%
21.0%
FL4-H:: CD42b-APC
3.60%
1.39%
77.7%
17.3%
16.8%
2.90%
77.9%
2.34%
12.6%
7.45%
68.6%
11.3%
0.627%
0.00%
76.9%
22.5%
Nonsynchronized MEG-01 without VPA
Nonsynchronized MEG-01 with VPA
Synchronized MEG-01 without VPA
Synchronized MEG-01 with VPA
(b)
CD41a+ Annexin V-
per 100,000 MEG-01 cells
Days
(c)
CD41a+ Annexin V+
per 100,000 MEG-01 cells
ns
Days
(d)
Nonsynchronized MEG-01
without TPO
MFI
Days
EVs
PLPs
(e)
Synchronized MEG-01
without TPO
CD41a
CD42b
(f)
Nonsynchronized MEG-01
with TPO
(g)
Synchronized MEG-01
with TPO

Supplementary Figure 4. PLPs and PEVs derived from synchronized MEG-01 cells express phosphatidylserine and platelet markers on their surface. (**a**) Analysis of PLPs and PEVs in the culture supernatant using flow cytometry. Cell debris was excluded using 1-μm beads. All particles with a size smaller than 1 μm were analyzed based on CD41a, CD42b, and phosphatidylserine exposure (annexin V). (**b**) The bar graph shows the number of CD41a-positive Annexin V-negative PLPs per 100,000 MEG-01 cells at days 7, 14, and 21 in the culture supernatant with or without VPA. (**c**) The bar graph shows the number of CD41a-positive and Annexin V-positive EVs per 100,000 MEG-01 cells at days 7, 14, and 21 in the culture supernatant with or without VPA. (**d-g**) Bar graphs of the mean fluorescence intensity of CD41a-positive CD42b-positive PLPs and EVs at days -1, -0.5, 0, 0.5, 1, and 2 in the culture supernatant. All experiments were performed more than 3 times, and the data represent the mean ± standard deviation. Statistical analyses were performed using ANOVA. ns, nonsignificance; **P* <.05, ***P* <.01, *** *P* <.001.

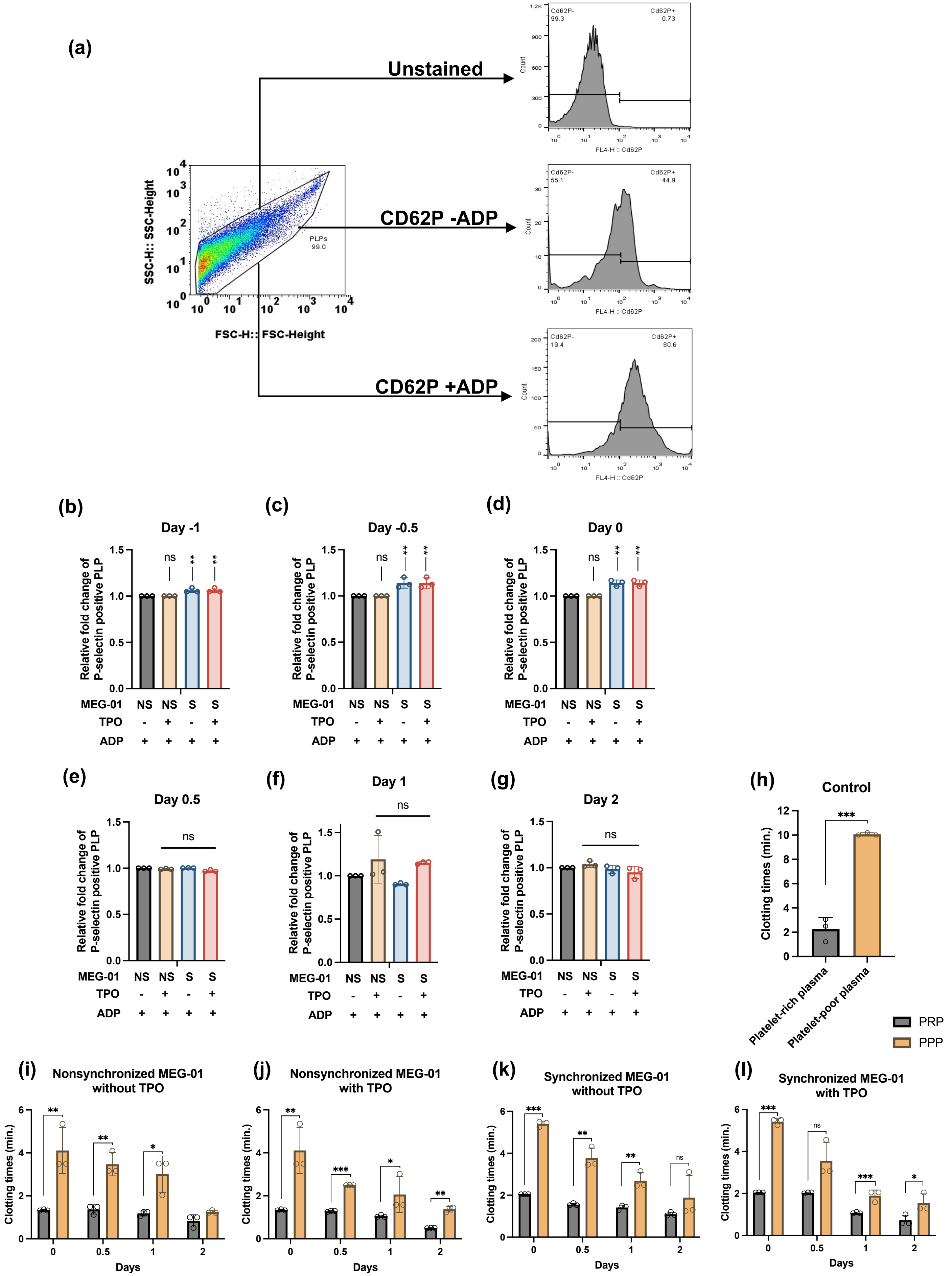

(a)
Unstained
CD62P -ADP
CD62P +ADP
SSC-H:: SSC-Height
FSC-H:: FSC-Height
PLPs
99.0
Cd62P-
99.3
Cd62P+
0.73
Cd62P-
55.1
Cd62P+
44.9
Cd62P-
19.4
Cd62P+
80.6
FL4-H :: Cd62P
Count
(b)
Day -1
(c)
Day -0.5
(d)
Day 0
(e)
Day 0.5
(f)
Day 1
(g)
Day 2
Relative fold change of P-selectin positive PLP
MEG-01 NS NS S S
TPO - + - +
ADP + + + +
ns
(h)
Control
Clotting times (min.)
Platelet-rich plasma
Platelet-poor plasma
PRP
PPP
(i)
Nonsynchronized MEG-01 without TPO
(j)
Nonsynchronized MEG-01 with TPO
(k)
Synchronized MEG-01 without TPO
(l)
Synchronized MEG-01 with TPO
Days

Supplementary Figure 5. Functional assay of PLPs derived from MEG-01 cells. (**a**) Analysis of P-selectin-positive PLPs when ADP was added to the culture supernatant using flow cytometry. (**b**-**g**) The bar graph shows the relative fold change in P-selectin-positive PLPs under different conditions when ADP was added per nonsynchronized MEG-01 cell without TPO and ADP at days -1, -0.5, 0, 0.5, and 2 (NS; nonsynchronized MEG-01 cells and S; synchronized MEG-01 cells). (**h**) The bar graph shows the clotting time of platelet-rich plasma (PRP), PPP (through a 0.1-μm filter) as a control, and culture medium under various conditions, including (**i**) nonsynchronized MEG-01 cells without TPO, (**j**) nonsynchronized MEG-01 cells with TPO, (**k**) synchronized MEG-01 cells without TPO, and (**l**) synchronized MEG-01 cells with TPO at days 0, 0.5, 1, and 2. The data are from 3 independent experiments. ns, nonsignificance; *$P < .05$, ** $P < .01$, *** $P < .001$.

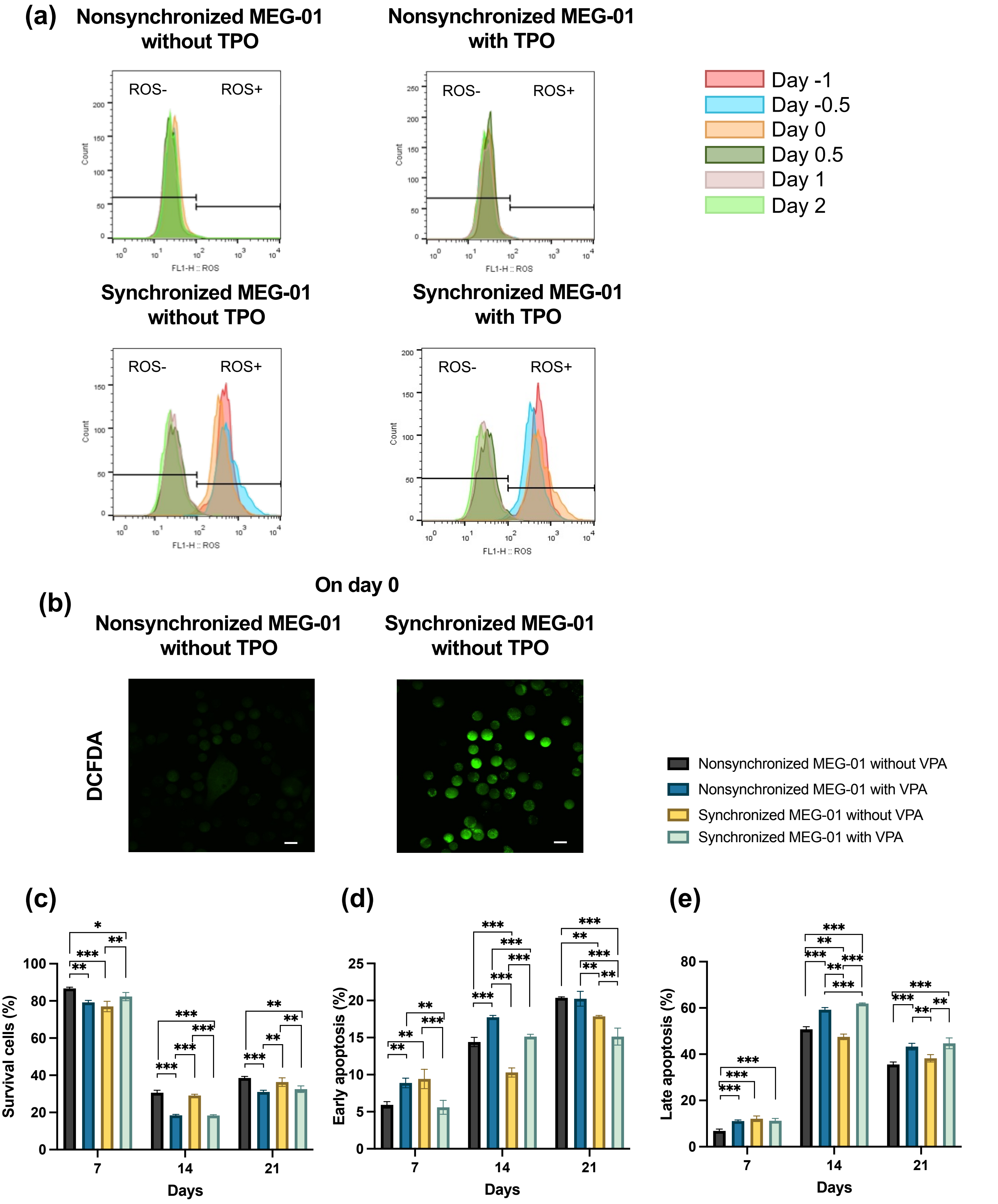

Supplementary Figure 6. Substantial changes in ROS and apoptosis in MEG-01 cells during synchronization and PLP formation. (a) Analysis of ROS measured using the DCFDA assay by flow cytometry under various conditions at days -1, -0.5, 0, 0.5, 1, and 2. (b) Confocal microscopy images of nonsynchronized and synchronized cells stained with a DCFDA cellular ROS detection assay kit according to the manufacturer's instructions (green) on day 0. ROS generation was captured using a 40x objective lens. The scale bar denotes 10 μm. The bar graph shows (c) the percentage of surviving cells, (d) early apoptosis, and (e) late apoptosis under various conditions with or without VPA on days 7, 14, and 21. The values represent the means ± SEs from 3 determinations per condition repeated 3 times (n=3). *P <.05, ** P <.01, *** P <.001.
